# Evaluating the Accuracy of Large Language Models in Selecting Appropriate Statistical Tests for Healthcare Research

**DOI:** 10.7759/cureus.111768

**Published:** 2026-06-29

**Authors:** Michael Paolella, Aditya Tadinada

**Affiliations:** 1 Oral and Maxillofacial Radiology, University of Connecticut Health, Farmington, USA

**Keywords:** artificial intelligence, healthcare research, large language models, statistical analysis, statistical test

## Abstract

Background: Appropriate statistical test selection is essential for producing valid and reproducible findings in healthcare research. However, many investigators encounter difficulties when determining the most suitable statistical methods for their study design and dataset. Recently, artificial intelligence-based large language models (LLMs) have emerged as potential tools capable of assisting researchers with methodological decisions, including statistical analysis.

Objective: The objective of this study was to evaluate whether artificial intelligence-based LLMs can accurately and reliably recommend appropriate statistical tests for healthcare research studies.

Methods: Four LLM platforms (ChatGPT (OpenAI, San Francisco, California, USA), Google Gemini (Google LLC, Mountain View, California, USA), Microsoft Copilot (Microsoft Corporation, Redmond, Washington, USA), and Grok (xAI, Palo Alto, California, USA)) and two traditional search engines (Google (Google LLC, Mountain View, California, USA) and Bing (Microsoft Corporation, Redmond, Washington, USA)) were evaluated. Forty published research articles were selected across four common study designs: systematic reviews, randomized controlled trials, cohort studies, and case-control studies (10 articles per category). Each article’s primary objective and statistical analysis were used to generate a standardized prompt describing the study scenario. These prompts were submitted to each LLM and search engine. Model responses were recorded and compared with the statistical tests used in the original studies. Accuracy was defined as agreement between the model’s recommendation and the statistical test used in the published article.

Results: LLMs demonstrated moderate-to-high accuracy in recommending appropriate statistical tests. Grok and Microsoft Copilot achieved the highest accuracy (34/40 correct recommendations), followed by Google Gemini (32/40) and ChatGPT (30/40). In contrast, traditional search engines (Google and Bing) did not provide statistical recommendations that matched the statistical tests used in the selected studies.

Conclusion: LLMs demonstrated promising capability in identifying appropriate statistical tests for healthcare research scenarios and outperformed traditional search engines in this task. While these findings suggest that LLMs may serve as useful decision-support tools for researchers, their recommendations should be interpreted cautiously and verified by individuals with statistical expertise. Continued evaluation of AI-based tools will be necessary to ensure their responsible integration into scientific research workflows.

## Introduction

Appropriate statistical test selection and rigorous evaluation of data are fundamental components of high-quality scientific research. The validity of study conclusions depends not only on data collection and experimental design but also on the correct application of statistical methods that align with the research question, data structure, and underlying assumptions [1]. Depending on the research question, every study has its own unique study design, and the right statistical test will evaluate the data in a certain specific way, so the data can be analyzed to glean the right answer. Inappropriate statistical analysis can lead to biased estimates, inflated error rates, misinterpretation of results, and ultimately unreliable or non-reproducible findings [2]. As concerns regarding research rigor and reproducibility continue to grow across scientific disciplines, the importance of sound statistical decision-making has become increasingly prominent [2].

Despite its critical role, statistical test selection remains a persistent challenge for many researchers. Scientific training programs often emphasize subject-matter expertise and experimental techniques while providing limited formal instruction in applied statistics. As a result, investigators may struggle to determine the most appropriate analytical approach when faced with variations in study design, sample size, variable type, distributional assumptions, or correlated data structures [3]. Common pitfalls include misuse of parametric tests when assumptions are violated, inappropriate handling of multiple comparisons, incorrect treatment of repeated measures, and confusion between exploratory and confirmatory analyses [4]. These issues are particularly prevalent among early-career researchers and clinicians, who may lack consistent access to biostatistical collaboration [4].

Although collaboration with biostatisticians is widely considered the gold standard for ensuring methodological rigor, access to such expertise varies considerably across institutions and healthcare settings worldwide. Time constraints, funding limitations, and the increasing volume of clinical research further compound these challenges [5]. Consequently, many investigators rely on statistical software defaults, online resources, or previously published examples when selecting analytical approaches. While convenient, these practices may not adequately address the methodological nuances of individual studies and may contribute to systematic analytic errors [5].

Recent advances in artificial intelligence, particularly large language models (LLMs), have introduced new possibilities for supporting clinical researchers in study design and data analysis. LLMs are capable of interpreting natural language descriptions of research questions, study designs, and datasets and generating tailored recommendations regarding statistical testing, assumptions, and interpretation [6]. Early investigations into AI-assisted research tools have demonstrated potential benefits in areas such as literature summarization, scientific writing, coding assistance, and clinical documentation [6,7]. Emerging work has also explored the potential role of LLMs in explaining statistical concepts and assisting with analytical planning in biomedical research [7].

However, existing studies in this domain have primarily focused on usability, explanatory capability, or general response accuracy rather than systematically evaluating whether LLMs can reliably select appropriate statistical tests for specific clinical research scenarios [8]. Additionally, concerns remain regarding model hallucination, inconsistency, and sensitivity to prompt phrasing, which may affect the reliability of AI-generated recommendations [9]. These limitations underscore the need for rigorous evaluation before widespread adoption of such tools in scientific research.

Currently, there is limited systematic evidence assessing the accuracy and reliability of LLMs in guiding statistical test selection for healthcare research studies. It remains unclear whether these models can appropriately account for study design, variable characteristics, and statistical assumptions in a manner comparable to trained biostatisticians [10]. Furthermore, the conditions under which LLMs may provide misleading or inappropriate statistical guidance have not been well characterized [9]. Addressing this knowledge gap is essential to determine whether AI-based language models can serve as reliable decision-support tools to enhance statistical rigor and reduce analytic errors in clinical research.

The objective of this study was to evaluate whether artificial intelligence-based LLMs can accurately and reliably recommend appropriate statistical tests for healthcare research studies.

## Materials and methods

Study design and selection criteria

The study was conducted at the University of Connecticut Health, Farmington, Connecticut, USA. This study employed a cross-sectional comparative design to evaluate the accuracy of four LLM platforms and two traditional search engines in recommending statistical tests for healthcare research. A total of 40 peer-reviewed research articles published between 2017 and 2025 were purposively selected to represent a diverse range of common healthcare study designs: systematic reviews/meta-analyses (n=10) focused on effect size estimation and heterogeneity; randomized controlled trials (n=10) evaluated therapeutic interventions and longitudinal outcomes; cohort studies (n=10) assessed risk factors and time-to-event data; and case-control studies (n=10) investigated associations and diagnostic accuracy. 

Model selection and prompt engineering

The following AI platforms and search engines were evaluated: LLMs: ChatGPT-5.5 (free) (OpenAI, San Francisco, California, USA), Google Gemini 3.5 Flash (free) (Google LLC, Mountain View, California, USA), Microsoft Copilot (free) (Microsoft Corporation, Redmond, Washington, USA), and Grok 4.3 (free) (xAI, Palo Alto, California, USA). Search engines included were Google (Google LLC, Mountain View, California, USA) and Bing (Microsoft Corporation, Redmond, Washington, USA). For each of the 40 articles, a standardized prompt was generated by extracting the primary study objective and the description of the data structure (e.g., variable types and longitudinal nature). To maintain consistency, the prompt followed a specific query format: "What type of statistical test would you run to [primary objective of the study]?” Data Collection and Evaluation. This question structure was used to give the AI platforms and search engines a specific, straightforward prompt. 

Prompts were submitted to each platform between 2025 and early 2026. Each prompt was run one time. Responses were recorded verbatim and compared against the "gold standard," defined as the actual statistical tests reported in the Materials and Methods section of the original published articles. This was done by a single reviewer, with a senior and experienced reviewer reviewing the initial set of outputs, and included periodic inputs and discussion. Accuracy was assessed using a binary scoring system. Correct (Score 1): The platform recommended the exact test or a statistically equivalent method used in the original paper. Incorrect (Score 2): The platform failed to mention the primary test, recommended an inappropriate method, or provided only generic search results/links without a specific recommendation. 

The answers from the AI platforms and search engines were copied and transferred to a Microsoft Word (Microsoft Corporation, Redmond, Washington, USA) document. Six total documents were created, one for each of the four AI platforms and one for each of the two search engines. Each document was separated into four sections based on the categories of articles used: systematic reviews/meta-analyses, randomized controlled trials, cohort studies, and case-control studies. Each section was divided into three subsections for each article used: the title of the article, the specific prompt that was searched, and the answer generated for the specific prompt. This was done to have a record of answers generated, which could be referred to during data analysis. 

The results of the answers compared to the "gold standard" were displayed using Microsoft Excel (Microsoft Corporation, Redmond, Washington, USA). Four sheets were included based on the categories of articles used: systematic reviews/meta-analyses, randomized controlled trials, cohort studies, and case-control studies. Within each sheet, headings were created, including the name of each article, its links, and they were numbered from one to ten. The articles were examined and analyzed for three main pieces of data that were also turned into headings: sample size, the primary objective of the article, which was used to help develop each specific prompt, and the statistical analysis that was used. The final headings in each sheet were the names of the AI platforms and the search engines. Under each of these final six headings was the prompt that was created and searched, as well as the answers that were generated, and whether they were correct. 

## Results

Overall performance of platforms

The LLMs demonstrated significantly higher accuracy compared to traditional search engines in identifying appropriate statistical tests. These results can be seen in Table 1. The total accuracy scores across all 40 scenarios were as follows: Grok and Microsoft Copilot achieved the highest performance, correctly identifying the statistical test in 34 out of 40 cases (85%). Google Gemini correctly identified tests in 32 out of 40 cases (80%). ChatGPT followed with 30 out of 40 correct recommendations (75%). Google and Bing failed to provide direct, correct statistical recommendations, often yielding incorrect results for all questions asked, and only provided general information or links rather than specific test selections. 

**Table 1:** Number of correct responses of each large language model and search engine for each article category ChatGPT: OpenAI, San Francisco, California, USA; Google Gemini: Google LLC, Mountain View, California, USA; Microsoft Copilot: Microsoft Corporation, Redmond, Washington, USA; Grok: xAI, Palo Alto, California, USA; Google: Google LLC, Mountain View, California, USA; Bing: Microsoft Corporation, Redmond, Washington, USA

# of Correct Responses	Randomized Controlled Trials	Cohort Studies	Case-Control Studies	Systematic Reviews
ChatGPT	8/10	8/10	8/10	6/10
Google Gemini	8/10	9/10	10/10	5/10
Microsoft Copilot	10/10	9/10	9/10	6/10
Grok	10/10	9/10	9/10	6/10
Google	0/10	0/10	0/10	0/10
Bing	0/10	0/10	0/10	0/10

Performance by study design

Randomized controlled trials saw the highest accuracy for some models, with Copilot and Grok achieving 100% (10/10) accuracy. ChatGPT and Gemini each correctly identified eight out of 10 tests, often correctly suggesting linear mixed-effects models and repeated measures ANOVA.

Cohort studies showed strong performance in identifying survival analysis (Cox proportional hazards) and regression models, with Gemini, Copilot, and Grok each achieving 9/10 accuracy.

The performance of case-control studies remained high, with Gemini achieving 10/10 accuracy, followed by Copilot and Grok at 9/10.

Systematic reviews were the most challenging category for LLMs. Accuracy dropped to 6/10 for ChatGPT, Copilot, and Grok, and 5/10 for Gemini. Common errors included suggesting general regression models instead of the specific random-effects models or reclassification indices (NRI/IDI) used in the source papers.

Search engine observations

Traditional search engines (Google and Bing), which can be viewed as secondary benchmarks in this study, consistently underperformed in direct test selection. While they often provided links to the correct article or statistical decision trees, they required the user to manually interpret the information to find the correct test, thus failing the "direct recommendation" criteria of this study. 

## Discussion

The present study evaluated the ability of artificial intelligence-based LLMs to recommend appropriate statistical tests for healthcare research scenarios. Statistical test selection represents a critical step in the research process, as inappropriate analytical methods can introduce bias, increase the likelihood of incorrect inferences, and undermine the reproducibility of scientific findings [1,2]. The results of this study demonstrate that contemporary LLMs exhibit a notable capacity to interpret research scenarios and identify appropriate statistical tests with moderate-to-high accuracy.

Among the models evaluated, Grok and Microsoft Copilot demonstrated the highest performance, correctly identifying statistical analyses in 85% of the scenarios examined. Google Gemini and ChatGPT also demonstrated relatively strong performance, although slightly lower than the top-performing models. These findings suggest that LLMs possess meaningful potential as decision-support tools capable of assisting investigators in preliminary statistical planning.

The contrast between LLM performance and traditional search engines observed in this study is particularly noteworthy. Search engines primarily rely on keyword indexing and information retrieval from existing webpages. While effective for locating relevant literature, they do not generate tailored analytical recommendations based on contextual descriptions of research designs. In contrast, LLMs are capable of synthesizing contextual information and applying learned statistical reasoning patterns when responding to natural language prompts. This capability likely explains the superior performance of LLMs observed in this study.

These findings align with emerging research indicating that LLMs may have significant utility in biomedical research workflows. Previous investigations have demonstrated that LLMs can assist researchers with literature review, manuscript drafting, coding for statistical software, and methodological explanation [6,7]. More recent work has explored the integration of LLMs into biostatistical workflows, suggesting that AI-assisted systems may support data analysis planning when used appropriately and under expert supervision [9].

Another important consideration when interpreting the results of this study is the influence of prompt design on model performance. Prior research has demonstrated that LLM outputs can vary depending on the phrasing, specificity, and contextual detail included in prompts [10]. This phenomenon, commonly referred to as prompt sensitivity, suggests that the reliability of AI-generated statistical recommendations may depend on how clearly the research scenario is described. Investigators using LLMs for methodological guidance should therefore provide detailed descriptions of study design, variable types, and outcome measures to improve response accuracy.

The findings of this study also highlight the potential role of LLMs as educational tools. Early-career researchers, clinicians, and trainees often encounter difficulty selecting appropriate statistical methods, particularly when formal statistical training is limited. LLM-based systems may provide accessible explanations and preliminary recommendations that help researchers better understand methodological considerations during the early stages of study design. However, these tools should be viewed as supplementary aids rather than replacements for formal statistical expertise.

Despite the promising findings of this study, several limitations must be acknowledged. First, the analysis included 40 research scenarios derived from four study designs. While these study types are common in medical literature, they do not encompass the full range of statistical techniques used in biomedical research. Many studies require more complex analytical approaches, including multivariable regression modeling, survival analysis, generalized linear models, and hierarchical or mixed-effects modeling. Future studies should examine the performance of LLMs in recommending these more advanced analytical techniques.

Second, the evaluation metric used in this study compared model recommendations with the statistical tests used in the selected published articles. While this approach provides a practical benchmark, it does not necessarily guarantee that the original statistical methods used in those studies were optimal. Previous analyses of biomedical literature have documented substantial rates of statistical misuse and methodological errors in published research [3,4]. Consequently, agreement with published analyses should not be interpreted as definitive evidence of methodological correctness. One of the other limitations of this study was that it had one reviewer scoring the outputs, and future studies can expand the scope by including repeated prompts and multiple reviewers.

Another limitation involves the inherent unpredictability of LLMs. AI systems may occasionally generate incorrect or fabricated responses, a phenomenon commonly described as "hallucination" [11,12]. Such inaccuracies highlight the importance of maintaining human oversight when applying AI-generated recommendations in scientific research contexts. Transparency and accountability of AI systems are hard and challenging topics being discussed worldwide, and it is important to construct such guardrails in today's AI age [13,14]. As discussed in a study, researchers should verify AI-generated statistical guidance using established methodological resources or consultation with trained biostatisticians [15].

Finally, the rapidly evolving nature of artificial intelligence technologies may influence the generalizability of the present findings. Differences in model architecture, training datasets, and update cycles may significantly affect model performance. As LLM technology continues to improve, future iterations may demonstrate substantially greater capability in statistical reasoning and methodological guidance.

## Conclusions

This study demonstrates that artificial intelligence-based LLMs show promising accuracy in recommending appropriate statistical tests for healthcare research scenarios. Several LLM platforms were able to correctly identify statistical analyses used in published studies with relatively high accuracy and substantially outperformed traditional search engines in this task.

These findings suggest that LLMs may serve as useful decision-support tools for researchers during the early stages of study planning and methodological development. However, the potential for inaccurate or inconsistent responses underscores the importance of maintaining appropriate human oversight. LLMs should therefore be considered complementary tools that support, rather than replace, formal statistical training and professional biostatistical consultation. Continued evaluation of AI-assisted research tools will be essential to ensure their responsible and effective integration into scientific research workflows.
